# Spatial and Temporal Hot Spots of *Aedes albopictus* Abundance inside and outside a South European Metropolitan Area

**DOI:** 10.1371/journal.pntd.0004758

**Published:** 2016-06-22

**Authors:** Mattia Manica, Federico Filipponi, Antonello D’Alessandro, Alessia Screti, Markus Neteler, Roberto Rosà, Angelo Solimini, Alessandra della Torre, Beniamino Caputo

**Affiliations:** 1 Dipartimento di Sanità Pubblica e Malattie Infettive, Università di Roma “Sapienza”, Piazzale Aldo Moro 5, Rome, Italy; 2 Dipartimento di Biodiversità ed Ecologia Molecolare, Centro Ricerca e Innovazione, Fondazione Edmund Mach, San Michele all'Adige, Trentino, Italy; 3 mundialis GmbH & Co. KG, Bonn, Germany; Liverpool School of Tropical Medicine, UNITED KINGDOM

## Abstract

*Aedes albopictus* is a tropical invasive species which in the last decades spread worldwide, also colonizing temperate regions of Europe and US, where it has become a public health concern due to its ability to transmit exotic arboviruses, as well as severe nuisance problems due to its aggressive daytime outdoor biting behaviour. While several studies have been carried out in order to predict the potential limits of the species expansions based on eco-climatic parameters, few studies have so far focused on the specific effects of these variables in shaping its micro-geographic abundance and dynamics. The present study investigated eco-climatic factors affecting *Ae*. *albopictus* abundance and dynamics in metropolitan and sub-urban/rural sites in Rome (Italy), which was colonized in 1997 and is nowadays one of the most infested metropolitan areas in Southern Europe. To this aim, longitudinal adult monitoring was carried out along a 70 km-transect across and beyond the most urbanized and densely populated metropolitan area. Two fine scale spatiotemporal datasets (one with reference to a 20m circular buffer around sticky traps used to collect mosquitoes and the second to a 300m circular buffer within each sampling site) were exploited to analyze the effect of climatic and socio-environmental variables on *Ae*. *albopictus* abundance and dynamics along the transect. Results showed an association between highly anthropized habitats and high adult abundance both in metropolitan and sub-urban/rural areas, with “small green islands” corresponding to hot spots of abundance in the metropolitan areas only, and a bimodal seasonal dynamics with a second peak of abundance in autumn, due to heavy rains occurring in the preceding weeks in association with permissive temperatures. The results provide useful indications to prioritize public mosquito control measures in temperate urban areas where nuisance, human-mosquito contact and risk of local arbovirus transmission are likely higher, and highlight potential public health risks also after the summer months typically associated with high mosquito densities.

## Author Summary

The tiger mosquito (*Aedes albopictus*) originates from tropical Asia, but over the last decades has invaded many regions worldwide. In several Southern Europe and US urban areas it has become the mosquito species creating the highest nuisance to the citizens, spoiling recreational outdoor activities by aggressively biting during daytime hours. Moreover, it has also become a public health concern due to its capability of transmitting to humans several exotic arboviruses. For an effective control strategy against these mosquitoes a profound knowledge of their preferences in terms of environment is mandatory in order to better understand what affects and determines their distribution. We analyzed some of these factors in the city of Rome and in its outskirts by collecting mosquitoes during an entire summer season. Results show that *Ae*. *albopictus* presence is more abundant in strongly urbanized areas compared to more green ones, but that within metropolitan Rome the highest densities are observed in small green areas within heavily urbanized settings, such as private gardens or public recreational areas, which should thus be the first target of anti-mosquito activities. Results also show that very high densities are not only observed in the warmest summer months as expected, but also in autumn, when people are less prepared to defend themselves from mosquito attacks.

## Introduction

The mosquito *Aedes* (*Stegomyia*) *albopictus* (Skuse) is classified among the 100 worst invasive species in the Global Invasive Species Database (http://www.issg.org/database/species) including all species of micro-organisms, fungi, plants and animals globally recognized as major threats to biodiversity and/or human activities. In the last 40 years, the species has been able to spread from its native range of distribution in rural tropical South-East Asia worldwide, largely through the transportation of its relatively cold-hardy and long-lived eggs via the international trade in used tires [1,2] and to the capacity to colonize temperate regions by photoperiodic egg diapause [1,3,4]. Another key element favouring *Ae*. *albopictus* expansion particularly to urban environments has been its ability to shift from natural larval habitats in forest edges (e.g. tree holes, bamboo stumps, and bromeliads) to anthropogenic containers (e.g. rain catch basins, tires, cemetery urns, vases, water storage containers) [4].

The first introductions of *Ae*. *albopictus* in Europe were documented in Albania in 1979 [5] and 10 years later in Italy [6], where it has become a permanent pest in most regions [7]. In recent years the species gradually spread into other Mediterranean countries, including France, Spain, Slovenia, Croatia, Bosnia and Herzegovina, Montenegro, and Greece [3]. Due to its aggressive daytime outdoor biting behaviour and to its ability to transmit a large variety of arboviruses [3], the species represents an important nuisance as well as a major public health concern in all non-native countries where it has established [8]. *Aedes albopictus* has been responsible for large chikungunya virus (CHIKV) epidemics in the Indian Ocean in 2005–2007 [9,10] and of a CHIKV outbreak in northern Italy in 2007 [11]. Since then it has been associated with autochthonous transmission of CHIKV and dengue virus (DENV) in France [12–15] and of DENV in Croatia [16]. Notably, these were the first autochthonous DENV cases reported in Europe since the outbreak in Greece in 1927–1928 caused by temporary establishment of a population of the tropical vector *Aedes aegypti* [17,18]. Models estimating climate change impact on spatio-temporal trends for risk exposure and season of transmission of CHIKV in Europe predict that Mediterranean regions will become increasingly climatically suitable for transmission, with highest risk of transmission by the end of the 21st century in France, Northern Italy and the Pannonian Basin (East-Central Europe) [19]. Moreover, a recent epidemic of Zika virus, with 440 000–1 300 000 estimated human cases in Brazil in 2015, is raising concerns about the risk of its introduction and local transmission by *Ae*. *albopictus* in Europe [20]. Finally, its opportunistic biting behavior [21,22] could involve *Ae*. *albopictus* in the transmission to humans of zoonotic pathogens such as West-Nile virus [23] and Dirofilaria canine nematodes [24,25].

Several authors used eco-climatic factors to predict the potential spatial distributions of *Ae*. *albopictus* and public health related threats. Most of these studies have focused on temperature to identify potential limits of the species range, indicating thresholds of minimum temperature in the coldest months and of heat accumulation [26–29] and producing maps to identify areas suitable for stable colonization [30–32]. Other studies exploited models to predict the species distribution using a broader range of climatic variables, including rainfall[1,31,33–38]. Recently, Kraemer et al. [39] developed an improved model combining climatic, environmental, land-cover and anthropogenic variables to predict the species probability of occurrence. Finally, Roche et al. [40] showed that human activities are particularly important for the species dispersion, while land use is a major factor for its establishment. Given the scale at which these studies were carried out, they are useful to predict *Ae*. *albopictus* future expansion and to improve surveillance programs by detecting the species introduction at its earliest stages when it is still possible to prevent its establishment [41].

However, in areas permanently colonized by *Ae*. *albopictus* it is crucial to identify potential spatial and temporal hot-spots of abundance which could be associated with higher nuisance biting and risk of disease transmission in order to prioritize mosquito control interventions. In fact, it has been demonstrated that treatment of hot-spot can be incorporated successfully into existing integrated mosquito management programs to increase their cost-effectiveness [42]. In general, availability of suitable breeding and resting sites along with the presence and abundance of competing species and of potential hosts (which in turn vary in relation to landscape composition, climatic conditions and host demography) are known to shape mosquito abundance at a local scale. In the absence of competition with *Aedes aegypti*, urbanization has been shown to favor high *Ae*. *albopictus* abundance [43,44] and landscape and human activities have been found to be crucial to predict its actual local distribution and relative abundance [45]. To date, the specific effects of these variables in shaping *Ae*. *albopictus* micro-geographic abundance and dynamics at temperate latitude in Southern Europe are poorly understood. The few studies carried out so far showed a positive association between host-seeking female abundance and temperatures and a negative one with rainfall in north-east Italy [46]. Additionally, a positive association between number of eggs and vegetation around ovitraps was detected in a small highly urbanized site within Rome [47].

The present study aims to investigate eco-climatic factors affecting *Ae*. *albopictus* abundance and dynamics in metropolitan versus sub-urban/rural sites in Rome (Italy), which was colonized by *Ae*. *albopictus* in 1997 and became one of the most infested metropolitan areas in Southern European temperate regions [48,49].

## Materials and Methods

### Mosquito sampling and study sites

**Fig 1:**
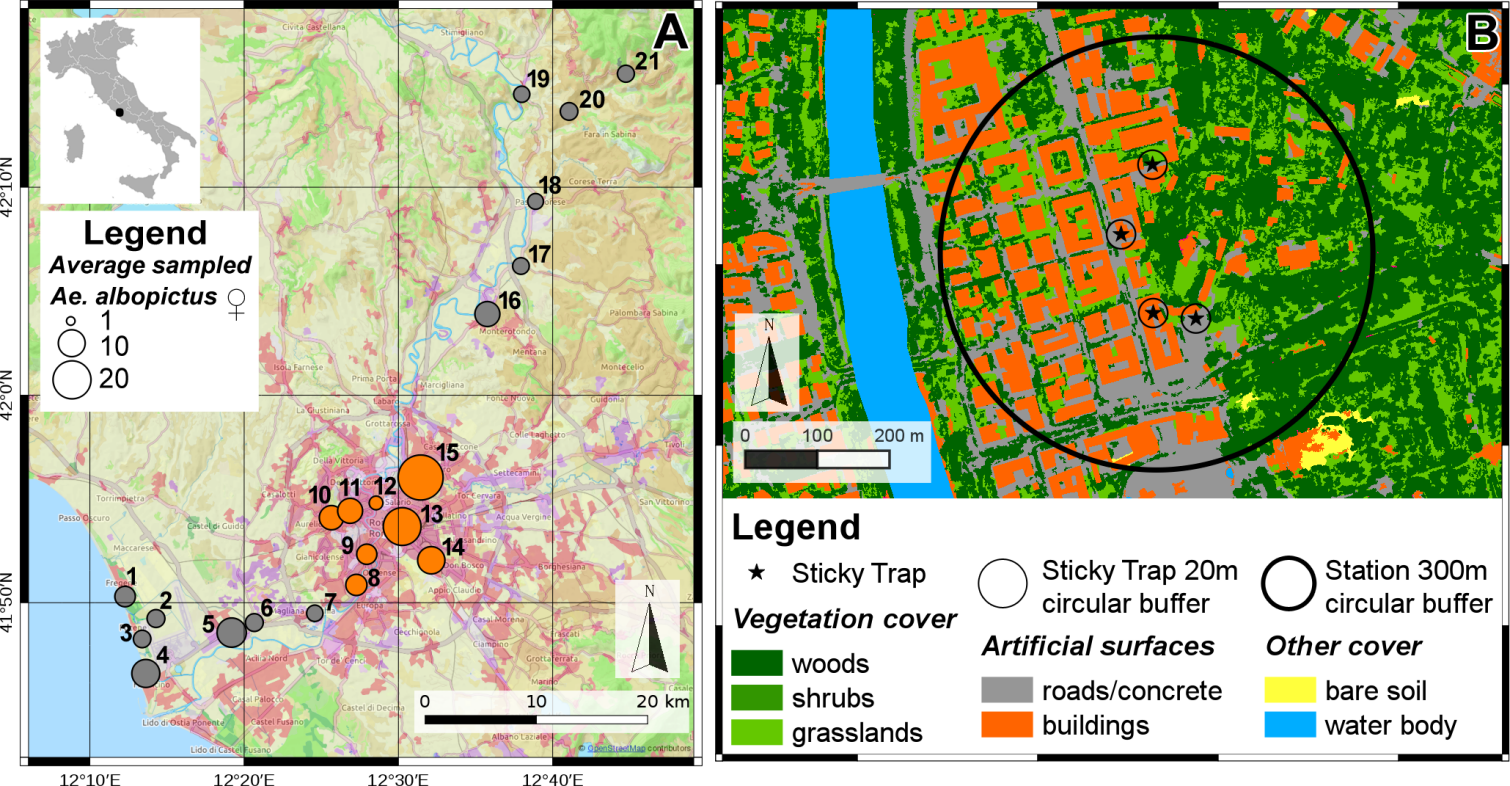
Mean abundance of *Aedes albopictus* collected along the 70 km-transect encompassing the metropolitan area of Rome. a) Map showing the weekly mean female abundance during the 18 sampling weeks in the 21 sampling stations (study sites); orange dot = “Metropolitan” site; grey dots = “Sub-Urban-Rural” site. b) Map showing the land cover variables in one of the 21 sampling sites, showing the 300 m-circular buffer calculated from the centroid of the convex hull generated from Sticky Traps (black star) and the 20 m-circular buffer around each Sticky Trap.

Twenty-one study sites (hereafter referred as stations) were selected along a 70 km-transect across and beyond the most urbanized and densely populated metropolitan area of Rome (Italy), corresponding to the train route from the coast to Appennino mountains (Fig 1). All sites were below 300m asl, with the exception of station 20 (330m asl) and 21 (460m asl). Groups of four sticky traps (STs, [50]) were located in a 300 m-radius area within each station (for a total of 84 STs), positioned on site and geo-referenced using GPS. The 300 m-radius was calculated from the centroid of the convex hull generated from groups of four neighbouring sampling points (i.e. STs) and corresponds to *Ae*. *albopictus* maximum dispersal range (i.e. 300 m; [51]). A 20 m circular buffer was calculated around each ST. This buffer at ST level corresponds to the largest one used in a similar study that showed an association between land cover variables and mosquito abundance [47]. Moreover, a 3 km-circular buffer was calculated from the centroid of the convex hull generated from groups of four neighbouring STs. Information obtained from the 3 km-circular buffer was used to assign each station to either metropolitan or suburban/rural area.

Sticky traps were monitored for 18 weeks from July 10^th^ to November 8^th^ 2012 on a weekly basis by substituting sticky panels with freshly glued ones and recording water leftovers. Trapped mosquitoes were morphologically identified and counted on site.

### Dataset description

A fine scale spatio-temporal dataset was built to analyze the effect of a set of climatic and socio-environmental variables on *Ae*. *albopictus* abundances in the 21 stations.

Open source software were used for the construction of the spatial dataset: GRASS GIS [52] for data processing and spatial analysis and QGIS [53] for spatial analysis and layout generation. The spatial dataset included time-dependent climatic variables and time-independent socio-environmental variables, as follows:

#### Climatic variables

Land Surface Temperature (LST) for each station was extracted from reconstructed temporal series of MODIS satellite data, collected by NASA (http://modis.gsfc.nasa.gov) and following methods described in Metz et al. (2014) [54]. LST data represent the estimation of temperature detected at earth surface by the satellite sensor, with gap-filled pixels at 250m spatial resolution. Daily minimum, maximum and mean LST were extracted. These variables were used to compute the mean of daily LST minimum, maximum, mean, and range for each trap over each sampling week in the period June–November 2012. Mean LSTs were computed not only for the sampling week (Lag 0), but also considering different time-spans in the weeks before the sampling in order to take into account the role of climate variables during the larval development in affecting *Ae*. *albopictus* adult abundance and survival. A total of 4 temporal windows (Lag 1–4) were computed over the 1–4 weeks preceding the sampling weeks. Subsequently, the mean LST from Lag 1 to Lag 4 were also considered as climate variables.

Growing Degree Days (GDD) were computed in order to take into account that heat accumulation influence *Ae*. *albopictus* life cycle and development. A value of 11° C was used as baseline temperature [4]. Moreover, the weekly-accumulated GDD and a bounded estimate of accumulated GDD was calculated as in Roiz et al. (2015) [55].

Daily rainfall maps were generated by spatial interpolation of daily rainfall data acquired in 67 meteorological sampling stations, collected by the Hydrographic Service of Regione Lazio and disseminated through the hydrographic annals (http://www.idrografico.roma.it/annali). Daily rainfall maps were used to compute the total rainfall in each trap location for each week of the period June–November 2012. The same temporal windows as for LST (from Lag 0 to Lag 4) were considered.

#### Socio-environmental variables

A land cover map was initially generated for the following 7 different classes retrieved from digital multispectral aerial imagery collected by optical sensor in the visible spectrum in 24 and 27 June 2008 at 0.5 m spatial resolution (Source: Italian National Geoportal, http://www.pcn.minambiente.it/GN/). Mapped land cover classes were ‘bare soil’, ‘roads/concrete’, ‘buildings’, ‘woods’, ‘shrubs’, ‘grasslands’, ‘water bodies’. Classification was performed using SMAP (Sequential Maximum A Posteriori) supervised classification [56], in GRASS GIS 7, which segments multispectral images using a spectral class model known as a Gaussian mixture distribution and using spectral mean and covariance parameters. The SMAP segmentation algorithm improves accuracy and resolution of urban mapping by segmenting the image into regions rather than segmenting each pixel separately. Classification products were spatially filtered in order to partially remove classification errors and noise. Satellite data were confirmed by field workers during the initial positioning of STs. Collinear cover classes (representing proportions inside the buffer fixed space) were finally merged in two main land cover groups: ‘artificial surfaces’ (including ‘roads/concrete’ and ‘buildings’) and ‘vegetation cover’ (including ‘woods’, ‘shrubs’ and ‘grasslands’). Only the latter one was used in the statistical analysis to avoid collinearity. Surface (in square meters) and cover percentage for the two main land cover classes were calculated for each station within: i) the 20 m-radius buffer around each ST, ii) the 300 m radius-buffer and iii) the 3 km radius-buffer. Population data was extrapolated from population 2011 census data (source http://gisportal.istat.it/). For the municipality districts encompassing the stations of the study area, recorded population has been divided by the area of the district in order to obtain the population density (inhabitants/km^2^).

The qualitative variable “environment” (Metropolitan or Sub-urban) was defined by evaluating the percentage of artificial surfaces within the 3 km-radius buffer and population density within the 300 m-radius buffer. Specifically, the environment around the centroid of the convex hull generated from groups of four neighbouring sampling points (i.e. ST) was defined “Metropolitan” when either the percentage of artificial surfaces exceeded 50% or the density of human population exceeded 6,000 human inhabitants per square kilometer, and “Sub-Urban/Rural” when neither these conditions were met.

### Statistical analysis

The relationship between socio-environmental and climatic variables and *Ae*. *albopictus* abundance was investigated through generalized linear mixed models (GLMMs) and generalized additive mixed models (GAMMs). Response variable was the weekly number of *Ae*. *albopictus* adult females collected in each STs. Time-dependent (climatic) and time-independent (socio-environmental) variables were included in the model as predictors. In addition, the variable Day of Year (DoY) was also considered to investigate the temporal pattern of the population dynamic during the sampling period. Scatterplots, conditional boxplots, variance inflation factor (VIF) and concurvity [57] were used to assess non-linear relationships and collinearity among variables.

#### Analysis of *Aedes albopictus* abundance during the entire sampling season

A Negative Binomial GLMM was carried out to assess the effect of socio-environmental variables on *Ae*. *albopictus* adult abundance. The following explanatory variables were modelled linearly and centred to aid interpretation of model results [58]: vegetation cover percentage computed at 20 meter-buffer around the STs, vegetation cover computed at 300 meter-buffer, environment, all the two-way interactions and the three-way interaction. Sampling stations were included as random effect to incorporate a dependency structure between observations taken by the four ST in the same station. However, the model residuals showed temporal patterns. Therefore, to account for the time varying abundance of *Ae*. *albopictus* dynamic time-dependent (climatic) variables were also considered. Preliminary analyzes were carried out to identify which climatic variables (i.e. temperature and rainfall) would be a feasible predictor of mosquito abundance and to identify its lagged effect (different temporal windows) on mosquito abundance. Although similar preliminary approaches are not advocated since they may result in *post hoc* hypothesis [59], this step was necessary due to high collinearity both among climatic variables and within time windows of each variable (e.g. Temperature: LST Lag 0–4, max-min-mean LST, weekly-accumulated GDD, estimate of accumulated GDD). Specifically, univariate Negative Binomial GLMMs were carried out in the preliminary analyzes to investigate the relationship between mosquito abundance and each climatic variable in turn, computed for all temporal windows considered (see M&M: Dataset description, climatic variables). Climatic variables were standardized by subtracting their means and dividing by their standard deviations. All preliminary models presented bimodal temporal patterns in the residuals. Therefore, Negative Binomial GAMMs were carried out. The rainfall variables, the temperature variables and the variable Day of the Year (DoY) were included in turn as penalized thin plate regression spline smoother. Models were ranked using the Akaike Information Criterion (AIC) [60] separately for both climatic variables (temperature and rainfall) and DoY. Only the significant variable with its temporal window producing the lowest AIC was considered for inclusion in subsequent full model. Following the preliminary analysis, the variable DoY was included as a penalized thin plate regression spline smoother in the model assessing the effect of socio-environmental and climatic determinants on *Ae*. *albopictus* adult abundance (hereafter referred to as full model). Therefore, to account for the time varying abundance of *Ae*. *albopictus* the full model resulted a negative binomial GAMM instead of the initial GLMM.

#### Analysis of the two *Aedes albopictus* high-abundance phases

Since *Ae*. *albopictus* adult seasonal dynamics showed a bimodal pattern, statistical models were carried out to investigate the major drivers of both high-abundance phases. Similar to the procedure followed by Roiz *et al*. (2015) [55], one dataset associated to mosquito dynamics around the first peak (Phase-1) and another one associated with the second peak (Phase-2) were extracted. First, to focus on the high abundance phases only, only the observations collected in sampling dates with a positive upper confidence limit of the DoY smoother were considered. Afterwards, sampling dates prior the local minimum of the DoY smoother were assigned to Phase-1 dataset, while observations after the local minimum were used in Phase-2 dataset. Two separate analyzes were carried out to assess if socio-environmental and climatic variables may differently affect mosquito abundance in Phase-1 and 2. Following the same approach used for the entire season analysis, also here the full model for each Phase was carried out after the preliminary analysis. Since no temporal pattern was found in the residuals of models, Negative Binomial GLMMs instead of GAMMs were carried out.

All analysis were carried out using R software version 3.1.3 [61] and packages glmmADMB [62], gamm4 [63] and plyr [64].

## Results

A total of 8,846 *Ae*. *albopictus* adult females and 1,932 males were collected by 84 STs in 21 sampling stations over the 18-week sampling period along a 70 km-transect across and beyond the highly urbanized area of Rome (Fig 1). All the following statistical analyzes were restricted to the female collections. The overall weekly mean of *Ae*. *albopictus* females catches was 7.6 (Standard Error, SE = 0.5) and 5.6 (SE = 0.2) in the Metropolitan and in Sub-Urban/Rural area, respectively (S1 Table). A great variability in mosquito abundance was observed among stations: an overall weekly mean of 19.0 (SE = 2.2) and 1.8 (SE = 0.3) mosquitoes/ST were collected in the most and least infested station, respectively. The mean percentages for Vegetation Cover around STs were 36.9% (ranging from 0% to 95.6%) and 46.5% (ranging from 28.5% to 68.5%) in the 20 m- and 300 m-radius buffer, respectively.

### Predictors of *Aedes albopictus* abundance during the entire sampling season

**Fig 2:**
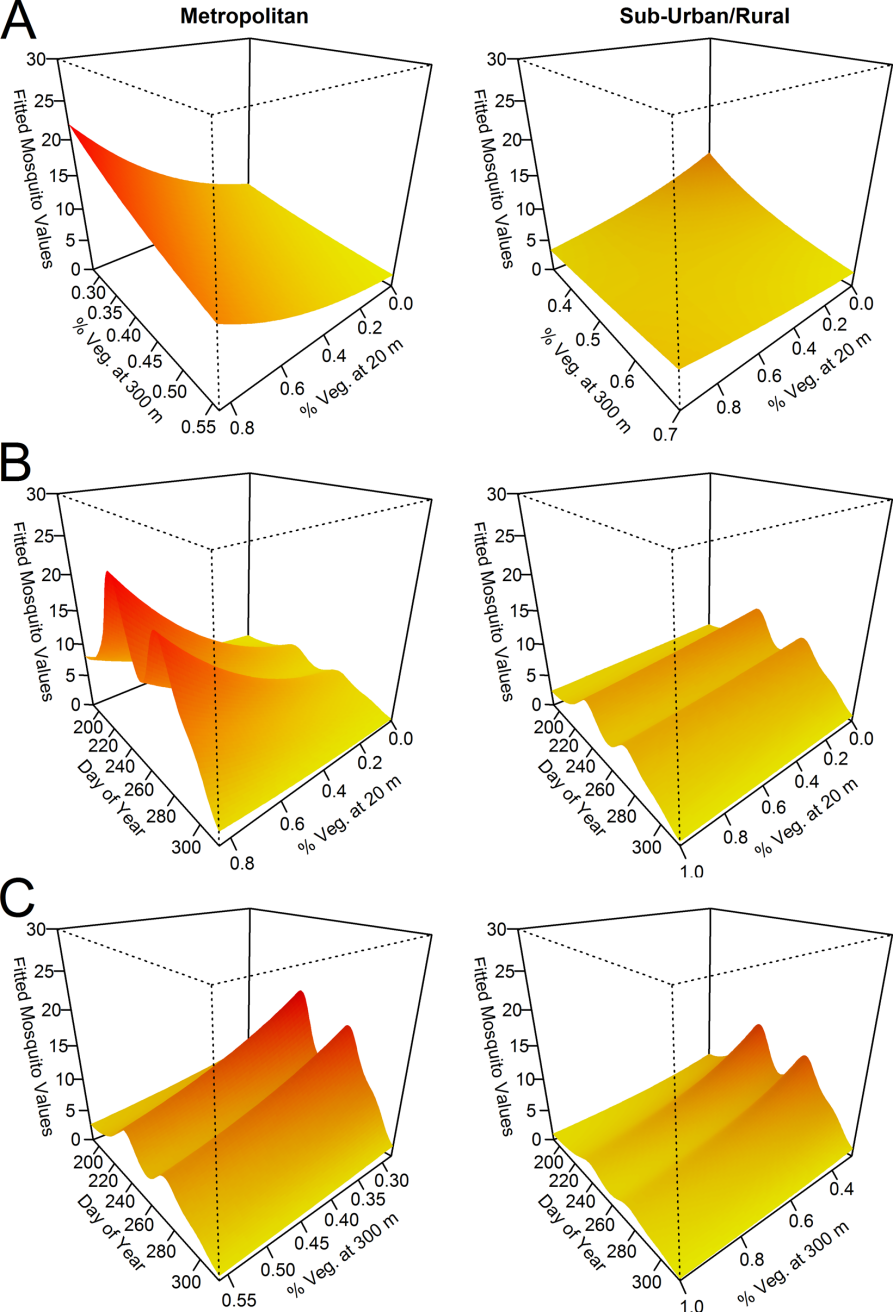
Fitted values (GAMM) of *Aedes albopictus* female abundance in metropolitan and sub-urban/rural environments in Rome. Left column = Metropolitan Environment; right column = Sub-Urban/Rural Environment. Fitted mosquito values (Z-axis) = fitted values of females/station/week. A: interaction between Vegetation Covers at 20 m and at 300 m buffers (scaled to the 0–1 interval) conditional to Days of the Year (DoY, considered at its mean values); B: interaction between Vegetation Cover at 20m and DoY conditional to vegetation cover at 300m (considered at mean values); C: interaction between Vegetation Cover at 300m and DoY conditional to Vegetation Cover at 20m (considered at mean values). Variables presented on the original scale (i.e. not centred).

Results of GAMMs showed that *Ae*. *albopictus* population dynamics was better modelled (lowest AIC with significant coefficients) by the Day of the Year (DoY) smoother than by time-dependent climatic predictors (i.e. temperature and rainfall) computed in different temporal windows (S2 Table). Therefore, DoY was taken as time-dependent variable in the full GAMM carried out to assess the effect of time-independent environmental predictors (i.e. Vegetation Cover) on *Ae*. *albopictus* abundance, and its role in Metropolitan vs Sub-Urban/Rural Environments. No multicollinearity among linear predictors was found (Variance Inflation Factor values < 2). On average, weekly *Ae*. *albopictus* abundance did not differ between Metropolitan and Sub-Urban/Rural environment (Table 1). However, a significant interaction term between Vegetation Cover and Environment was found in the 20 m-radius buffer, meaning that an increase of the proportion of Vegetation Cover was positively associated with *Ae*. *albopictus* abundance in the Metropolitan Environment, but not in the Sub-Urban/Rural one (Table 1, Fig 2). On the other hand, an increase of the proportion of Vegetation Cover in the 300 m-radius buffer was negatively associated with mosquito abundance in both Environments (Table 1).

**Table 1:** Results of GAMM of *Aedes albopictus* female abundance in metropolitan vs. sub-urban/rural environments.

Variable	Coeff.	SE	z-value	Pr(>|z|)
Intercept	1.562	0.142	11.009	2e^-16^ ***
Vegetation 20 m	2.059	0.271	7.597	3.03e^-14^ ***
Vegetation 300 m	-3.147	1.201	-2.619	0.009 **
Environment (Sub-Urban/Rural)	-0.039	0.175	-0.222	0.824
Vegetation 20 m * Vegetation 300 m	1.200	2.214	0.542	0.588
Vegetation 20 m * Environment (Sub-Urban/Rural)	-2.470	0.342	-7.226	4.96e^-13^ ***
Vegetation 300 m * Environment (Sub-Urban/Rural)	0.898	1.440	0.623	0.533
Vegetation 20 m * Vegetation 300 m * Environment (Sub-Urban/Rural)	5.015	2.734	1.834	0.067

Metropolitan Environment as reference level. Number of observation = 1353, number of stations = 21. Standard deviation of random effects = 0.33. Value of dispersion parameter = 1.8. The model included a smoothing term with 8 estimated degrees of freedom (approximate p-values <0.0001). Significance code: *** <0.001, 0.001<**<0.01, 0.01<*<0.05.

**Fig 3:**
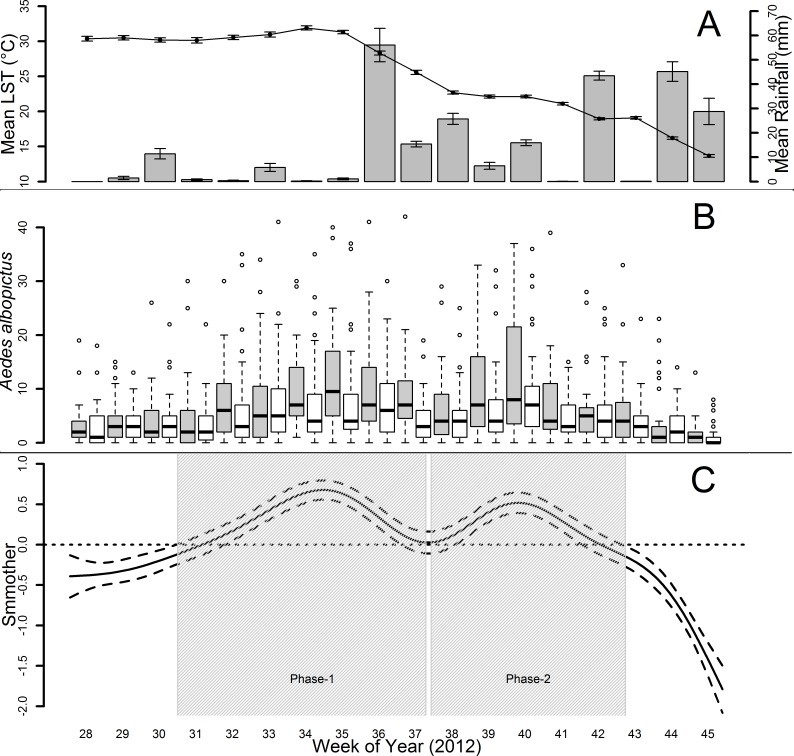
Temporal dynamic of *Aedes albopictus* females during 18 week-sampling in Rome. A: Temperature (LST, °C) and Rainfall (mm) observed temporal dynamics. Line graph = T, error bars = 95% confidence intervals, y-axis (left) = mean value of LST/week (Lag 0); bar graph = Rainfall, error bars = 95% confidence intervals, y-axis (right) = mean value of mm of rainfall/week (Lag 0). B: Observed mosquito temporal dynamics. Y-axis = boxplot of mosquito/week in Metropolitan (grey boxes) and Sub-Urban/Rural (white boxes) Environments. Boxes = first and third quartiles (the 25th and 75th percentiles). Line inside the box = median. The upper whisker extends from the boxes to the highest value that is within 1.5 * IQR (inter-quartile range: the distance between the first and third quartiles, so the height of the boxes). The lower whisker extends to the lowest value within 1.5 * IQR. Empty circles = outliers. C: Day of Year smoother (GAMM). Grey areas = phases exploited to investigate the climate drivers of the two peaks of mosquito abundance. X-axis = 18 weeks of collections in 2012.

Fig 3A shows temperature and rainfall during the 18 week-sampling period. Fig 3B shows the observed *Ae*. *albopictus* temporal dynamics in Metropolitan and Sub-Urban/Rural Environments. The shape of the estimated smoother (Fig 3C) shows bimodal mosquito population dynamics in both environments (no differences in the shape of the estimated smoothers in the two Environments was found), characterized by a first peak during the last weeks of August (weeks 34 and 35) and a second peak during the first week of October (week 40).

### Predictors of *Aedes albopictus* during each of the two high-abundance phases

The two phases (Phase-1 and -2) of highest mosquito abundance (in grey in Fig 3C) were analyzed separately in order to assess their respective climatic drivers. Results of univariate models—carried out in the preliminary analysis to assess which time-dependent variables would be the best predictor of mosquito abundance and to identify their lagged effect (different temporal windows)—showed that the accumulated LST at Lag 1 and the accumulated rainfall at Lag 3 were the most informative climatic predictors related to Phase-1 (S3 Table). However, when included in the full model with time independent variables, only LST at Lag 1 remained significant (Table 2). On the other hand, the accumulated rainfall at Lag 4 and the mean LST at the week of sampling (i.e. Lag 0) were the most informative climatic predictors in the preliminary analysis for Phase-2 (S3 Table). However, when they were included in the full model, only the accumulated rainfall at Lag 4 remained significant (Table 2). Socio-environmental time-independent variables in both models carried out for the two high abundance phases showed the same effects observed in the model carried out for the entire sampling season (Table 1). Specifically, a significant interaction term was detected between Vegetation Cover and Environment at 20 m-radius buffer, meaning that an increase of the proportion of Vegetation Cover was positively associated with *Ae*. *albopictus* abundance in the Metropolitan Environment, but not in the Sub-Urban/Rural one (Table 2). On the other hand, in both high-abundance phases an increase of the proportion of Vegetation Cover in the 300 m-radius buffer was negatively associated with mosquito abundance in both Environments (Table 2).

**Table 2:** Results of GLMM of *Aedes albopictus* female abundance in metropolitan vs. sub-urban/rural environments during the first and second phases of highest abundance.

GLMM Phase-1			GLMM Phase-2		
Variable	Coeff.	Pr(>|t|)	Variable	Coeff.	Pr(>|t|)
Intercept	1.858	2e^-16^ ***	Intercept	1.853	2e^-16^ ***
Rainfall Lag 1	-0.0004	0.871	Rainfall Lag 4	0.007	3.7e^-7^ ***
LST Lag 1	0.127	0.0008 **	LST Lag 0	0.030	0.355
Vegetation 20 m	1.906	2.5e^-6^ ***	Vegetation 20 m	2.030	3.1e^-5^ ***
Vegetation 300 m	-3.129	0.020 *	Vegetation 300 m	-3.673	0.021 *
Env. (Sub-urban/Rural)	0.045	0.822	Env. (Sub-urban/Rural)	-0.256	0.270
Veg. 20 m * Veg. 300 m	-2.027	0.536	Veg. 20 m * Veg. 300 m	1.650	0.661
Veg. 20 m * Env.	-2.186	1.6e^-5^ ***	Veg. 20 m * Env.	-3.080	4e^-7^ ***
Veg. 300 m * Env.	1.431	0.372	Veg. 300 m * Env.	2.912	0.125
Veg. 20m*Veg. 300m*Env.	5.960	0.130	Veg. 20m*Veg. 300m*Env.	6.841	0.139

Phase-1: number of observation = 543, number of stations = 21, standard deviation of random effects = 0.35. The model included a smoothing term with 8 estimated degrees of freedom (approximate p-values <0.0001). Phase-2: number of observation = 396, number of stations = 21, standard deviation of random effects = 0. 42. The model included a smoothing term with 8 estimated degrees of freedom (approximate p-values <0.0001). Significance code: *** <0.001, 0.001<**<0.01, 0.01<*<0.05.

## Discussion

The results of the analysis of the spatial distribution and relative abundance of *Ae*. *albopictus* along the 70 km-long transect encompassing the metropolitan area of Rome highlighted a complex relationship between landscape composition and mosquito abundance.

When focussing the analysis to the scale of the estimated flight range of the species [51]—corresponding in the experimental set up to a 300 m-buffer within each sampling station—results showed that high adult abundance was on average associated with highly anthropized habitats (rather than with highly vegetated ones), in both the Metropolitan and the Sub-Urban/Rural areas. This is consistent with characteristics of highly anthropized habitats which favour the mosquito life-cycle, such as high human population density providing more opportunities for blood-feeding and larger numbers of artificial water containers (such as flowerpots, rain catch basins, abandoned tires, and disposable tins) suitable for container-breeding mosquitoes. Moreover, especially in temperate regions, the replacement of natural soil and vegetation with artificial surfaces is known to elevate temperatures and alter rainfall regimes with respect to surrounding regions [65–67], favoring mosquito development and gonotrophic cycle [36]. Indeed, *Ae*. *albopictus* has been shown to reach very high densities in highly anthropized areas, in particular in the absence of sympatric competing *Ae*. *aegypti* populations [43,44,68].

On the other hand, when the analysis was restricted to a 20 m-scale in order to focus on the landscape factors at sticky trap level (as in [47]), a different pattern of mosquito abundance was observed depending on the location of the traps in Metropolitan or in Sub-Urban/Rural areas. In fact, while in the latter, the vegetation coverage at 20 m-scale did not affect *Ae*. *albopictus* abundance, in the Metropolitan area, sticky traps positioned within highly vegetated 20 m-buffers collected higher number of mosquitoes, especially when located in highly anthropized stations. Overall, results suggest that hotspots of *Ae*. *albopictus* abundance within the highly anthropized Metropolitan stations are associated with “small green islands”. Since the sticky traps used for mosquito sampling mostly collect ovipositing females as well as resting adults [22], these “small green islands” may represent ideal sites in which the mosquito founds optimal conditions to lay eggs and rest. Moreover, it can be speculated that “small green islands” are associated with higher abundance of potential resting and larval sites characterized by more suitable temperatures for larval development (as shown in New Jersey, US [69]). Finally, “small green islands” in highly anthropized Metropolitan areas (such as children playgrounds, elderly people meeting places, small private gardens and condominium gardens) may be also attractive for host-seeking females, as they are largely exploited by people for outdoor activities especially during late summer afternoons when the species is most active. In fact, from an epidemiological perspective, it would be relevant in the future to extend the analysis in order to represent spatial heterogeneities not only in mosquito abundance but also in mosquito-to-host ratio [45].

The results of the analysis of the *Ae*. *albopictus* seasonal dynamics showed a bimodal pattern (with a peak of abundance in August and one in October) in most sampling stations, revealing that the species may unpredictably reach very high abundance also after the summer season. The first peak in August was clearly temperature-driven, while rainfall accumulated in September (average value of 103 mm) following a month with very low rainfalls (average value in August = 7.4 mm) seems to be the major driver of the second peak unexpectedly observed in mid-October. Afterwards, when temperatures and photoperiod became sub-optimal for the species life cycle, the mosquito abundance rapidly decreased despite frequent rainfall. The first peak in August followed by a decrease in mosquito abundance in the following weeks is consistent with data from the early stage of the species colonization of Rome [70,71], as well as from subsequent years when the species had already become an established urban pest [50,72]. The second peak observed in the present work is less consistent with data reported in the past, although a second increase in the population abundance was frequently shown to occur in October, probably due to relatively high temperatures and rainfall accumulating after a few dry summer weeks, a common climatic pattern in Rome at least in the last 10 years (S1 Fig). Moreover, subsequent data from human-landing catches carried out in Rome confirm a bimodal seasonal dynamics with very high host-seeking mosquito abundance in the final part of the reproductive season (Manica et al. in preparation).

Some aspects of the experimental design deserve discussion. First, it may be argued that the seasonal-long trapping effort influenced mosquito abundance in the study sites. However, results from previous work clearly showed that only a small fraction of wild *Ae*. *albopictus* females is collected by STs even in the frame of a much more intense sampling scheme than the present one [51]. Second, it is conceivable that competition of sticky traps with other potential oviposition sites likely non-homogeneously distributed in space and time may have created a bias in the comparison of mosquito abundance among sampling sites and among different phases of the season. Unfortunately, every collection approach may suffer of some kind of bias. Given appropriate resources, it would have been beneficial to monitor potential breeding sites during the field activities. Third, the results refer to ovipositing/resting females and may not directly reflect mosquito/human ratio, which is a more relevant epidemiological parameter to be assessed by the recording abundance of host-seeking females. Finally, inferences on the landscape determinants of the spatial and temporal distribution of *Ae*. *albopictus* abundance here presented are aggregated (i.e. vegetation cover includes trees, grass, bushes, etc.) to increase statistical power and do not take into account the land use associated with human activities.

Despite these study limitations, the results allow relevant speculations from a public health perspective. First, the analysis of the climatic determinants of *Ae*. *albopictus* seasonal dynamics highlights how the association of permissive photoperiod and temperatures associated with rainfall at the end of the summer period may result in a second phase of high *Ae*. *albopictus* abundance. This is likely to occur not only in the Rome area, but also in other Mediterranean regions colonized by the species and showing a similar climatic pattern. Roiz et al. [55] showed that an extreme rainfall event increased and extended the species abundance in Montpellier (coastal France) leading to at least 11 cases of autochthonous CHIKV transmission. This led the authors to propose that mosquito control campaigns must be implemented after such heavy rainfall events. Our results extend this concept, as they suggest that also less extreme and repeated rainfall after a relatively long dry period (which characterized Rome in the past years and is predicted to became a typical scenario in Italy in future years due to climate changes [73]), may cause the replenishment of peridomestic containers where desiccated eggs of *Ae*. *albopictus* are present, giving rise to increased mosquito abundance a few weeks later. This implies that in South European areas, characterized now or in the future by a similar climatic pattern, monitoring and control campaigns should be planned also after the end of the summer season to prevent a possible second peak of the mosquito population abundance and its associated health threats. Second, our spatial analysis emphasizes the need to prioritize public mosquito control activities in “small green islands” within highly anthropized metropolitan settings (such as children playgrounds or elderly people meeting places) where nuisance, human-mosquito contact and risk of local arbovirus transmission are likely to be higher [74,75]. On the other hand, the study suggests that such a prioritization strategy might be ineffective outside the metropolitan areas, where no hot-spots of mosquito abundance have been identified.

## Supporting Information

S1 Table Weekly mean of *Aedes albopictus* adult females (±SE) in each of the 21 sampling station over 18-week sampling along a 70km-transect encompassing Rome metropolitan area. Each sampling station is characterized by population density (i.e. inhabitants/km^2^) and vegetation cover (i.e. percentage of areas covered by “vegetation” vs “artificial surfaces”) at a 300 m and 3 km radius areas. NA = not available.
(PDF)

S2 Table Result of Generalized Additive Mixed Models (GAMMs) of time-dependent climatic predictors during the whole sampling season. Rainfall variables were modelled in turn either non-linearly or linearly with the inclusion of a Day of Year (DoY) smoother in each model. Temperature (T) variables were modelled in turn non-linearly (GDD = Growing Degree Days; LST = Land Surface Temperature). For T variables, the DoY smoother was not included due to high collinearity (i.e. concurvity) between T and DoY. s() denotes the smoother term.
(DOCX)

S3 Table Result of Generalized Linear Mixed Models (GLMMs) of time-dependent climatic predictors during the two high *Aedes albopictus* abundance phases. (GDD = Growing Degree Days; LST = Land Surface Temperature).
(DOCX)

S1 Fig Climatic pattern in Rome (2003–2014). Data collected by the Hydrographic Service of Regione Lazio and disseminated through the hydrographic annals (http://www.idrografico.roma.it/annali). Meteorological sampling stations of Roma Sud. Upper panel: whole year data, Lower panel: highlight week 28–45 from whole year data.
(PDF)
