# Supplementary material for: Spatial and Temporal Hot Spots of Aedes albopictus Abundance inside and outside a South European Metropolitan Area
Source: PLoS Negl Trop Dis. 2016 Jun 22;10(6):e0004758. doi: 10.1371/journal.pntd.0004758 (PMC4917172; doi:10.1371/journal.pntd.0004758)
Supplement: S1 Table — Each sampling station is characterized by population density (i.e. inhabitants/km2) and vegetation cover (i.e. percentage of areas covered by “vegetation” vs “artificial surfaces”) at a 300 m and 3 km radius areas. NA = not available. (PDF) [file pntd.0004758.s001.pdf]

	GPS							
**Station ID**	**Latitude**	**Longitude**	**Ecology**	**Population Density**	**% Vegetation 3 km**	**% Vegetation 300 m**	**1**	**2**
1	41.83824382	12.2046277	Sub-Urban/Rural	1706.325	31.19	41.97	0.25 + 0.25	3.25 + 1.18
2	41.82066696	12.23801976	Sub-Urban/Rural	11.368	34.25	45.99	2.75 + 1.89	3.25 + 0.25
3	41.8042066	12.2230366	Sub-Urban/Rural	2696.952	28.65	33.39	2 + 1.68	6.33 + 1.2
4	41.7763722	12.22691256	Sub-Urban/Rural	4959.511	29.4	38.94	8.33 + 5.04	5.75 + 1.75
5	41.80934886	12.31985942	Sub-Urban/Rural	20.459	51.83	37.56	7.75 + 1.89	9 + 1.68
6	41.8174563	12.34435483	Sub-Urban/Rural	1820.085	61.54	44.58	3 + 0.58	1.75 + 0.48
7	41.82486978	12.40949916	Sub-Urban/Rural	21.425	65.58	56.86	2.67 + 2.19	2.5 + 2.18
8	41.84758002	12.45450048	Metropolitan	9298.187	57.15	50.6	5 + 4.67	6.67 + 4.26
9	41.87240106	12.465888	Metropolitan	18263.52	53.21	35.46	1.75 + 0.85	1.25 + 0.75
10	41.90178235	12.42769521	Metropolitan	16844.138	60.12	28.73	3.33 + 0.33	6.75 + 2.56
11	41.90746141	12.44775396	Metropolitan	23260.09	54.6	30.42	1.25 + 0.63	2 + 0.71
12	41.91353299	12.47583383	Metropolitan	3117.546	47.45	55.91	2.25 + 1.31	2 + 1.41
13	41.89463452	12.50397023	Metropolitan	26655.809	39.48	28.52	4 + 3	2.25 + 0.85
14	41.86761525	12.53541125	Metropolitan	10817.479	62.13	46.98	5.25 + 2.87	3 + 1.15
15	41.93417412	12.52382362	Metropolitan	12483.623	59.11	51.03	NA	10.5 + 2.6
16	42.06513769	12.59593012	Sub-Urban/Rural	5786.351	61.81	36.38	NA	7
17	42.10367001	12.63226228	Sub-Urban/Rural	32.283	67.85	64.46	NA	3.75 + 1.25
18	42.15555727	12.6482546	Sub-Urban/Rural	2372.94	62.65	47.15	NA	3.5 + 0.96
19	42.24127405	12.63323956	Sub-Urban/Rural	833.476	72.73	67.75	NA	1 + 0.58
20	42.22748119	12.6844347	Sub-Urban/Rural	285.649	75.04	68.46	NA	NA
21	42.25766386	12.74593705	Sub-Urban/Rural	562.593	82.71	65.73	NA	0.33 + 0.33

Table S1 – Weekly mean of *Aedes albopictus* adult females (±SE) in each of the 21 sampling station over 18-week sampling along a 70km-tra

							Weeks
**3**	**4**	**5**	**6**	**7**	**8**	**9**	**10**
3 + 1.53	2.5 + 1.5	6.25 + 2.95	10.5 + 3.88	8 + 3.72	8.75 + 3.12	8 + 2.38	7 + 4.02
8.5 + 2.25	6.25 + 1.65	13.75 + 7.7	10.75 + 4.8	5.25 + 1.65	9 + 3.24	6.5 + 2.9	1 + 0.41
4 + 1.58	3.67 + 2.19	2.5 + 0.87	7 + 2.68	3.25 + 0.48	2.75 + 0.95	2.75 + 0.63	0.75 + 0.25
5 + 1.47	4 + 1.63	6.75 + 3.09	9.25 + 1.49	15 + 3.54	16 + 3.89	13.75 + 3.52	9.5 + 2.25
4.5 + 1.44	4.75 + 2.32	14.75 + 6.76	20.5 + 7.9	35.5 + 8.72	17 + 7.84	12.25 + 2.29	NA
3 + 0.71	4 + 2.27	8.75 + 3.12	6.75 + 1.8	7.5 + 1.26	6 + 4	5.75 + 1.25	11
4 + 3.67	4.25 + 2.1	6.5 + 3.1	1.75 + 0.48	5.75 + 2.46	2 + 0.91	1.75 + 1.75	NA
4 + 1.15	4.5 + 2.9	6.5 + 2.9	5.75 + 2.56	4.75 + 1.65	10.25 + 4.99	5.5 + 4.5	NA
3.33 + 1.2	1 + 0.58	5.75 + 2.53	5 + 1	6.5 + 0.29	9.25 + 1.89	4.33 + 0.88	NA
5 + 1.96	5.75 + 2.1	5.75 + 2.46	4 + 1.91	7 + 1.87	12.75 + 3.64	7 + 2.97	6.75 + 1.49
3.5 + 0.96	3 + 1.08	7.33 + 1.2	1 + 0.41	11 + 3.34	10.25 + 3.09	9.25 + 2.14	6.25 + 2.02
0.75 + 0.75	1 + 0.58	2.67 + 1.76	3.25 + 2.29	3 + 1.15	3.5 + 1.26	1.75 + 1.44	2.75 + 1.8
3 + 2.04	10.5 + 5.33	16.25 + 6.06	11 + 3.14	29.75 + 13.55	40 + 15.58	13.5 + 5.2	19.25 + 7.91
0.75 + 0.48	1.75 + 1.11	3.75 + 0.85	5.25 + 2.66	10.5 + 3.01	7.75 + 2.78	6.75 + 2.69	9.5 + 3.84
13 + 4.93	11.33 + 9.4	10 + 3.63	25.75 + 3.57	20 + 3.19	43.75 + 22.61	41.5 + 16.82	10.75 + 1.03
8.5 + 4.63	8.33 + 6.89	10.5 + 10.5	3 + 0.58	10 + 4.51	22 + 14	16.5 + 6.22	5 + 1.53
3.5 + 0.96	3.25 + 1.49	5.75 + 1.11	5 + 1	4.75 + 1.18	6.5 + 2.47	3.5 + 1.55	2.67 + 0.33
4.67 + 2.6	3.33 + 1.45	4.25 + 1.97	5 + 2.68	5.5 + 1.55	5.5 + 2.22	3.25 + 0.25	3.67 + 0.33
3.25 + 1.97	2.25 + 1.31	2 + 1	4.5 + 2.84	7 + 4.42	5.25 + 3.64	6 + 2.35	4.33 + 2.33
2.25 + 0.48	3 + 0.71	1.5 + 0.29	10.5 + 3.66	4.5 + 2.53	5.25 + 1.25	7.25 + 1.65	5.25 + 2.29
1.33 + 0.33	1 + 0.41	0.25 + 0.25	2.75 + 0.85	5 + 1.08	9.25 + 4.27	8.25 + 1.11	5 + 1

nsect encompassing Rome metropolitan area. Each sampling station is characterized by population density (i.e. inhabitants/km^2) and vegetation cover (i.e.

11	12	13	14	15	16	17	18
3.5 + 1.76	6.5 + 2.06	13.5 + 6.38	6.25 + 1.8	10 + 4.71	2.5 + 0.96	3.5 + 2.25	0.75 + 0.48
0.5 + 0.29	2.75 + 1.49	1 + 0.71	2 + 0.41	3 + 1.53	1 + 0.71	0.25 + 0.25	0
3.25 + 1.6	5.25 + 2.59	15.25 + 4.66	3.5 + 1.66	3.75 + 1.03	6.25 + 1.31	2 + 0.71	1 + 0.41
11.5 + 4.73	16.75 + 10.53	24.75 + 9.2	11.25 + 2.5	17.75 + 3.68	7 + 1.08	8 + 1.15	3.25 + 1.11
9.25 + 3.71	12.75 + 7.16	12.5 + 3.28	5.33 + 1.33	8 + 2.86	11.5 + 4.33	8 + 2.04	4.67 + 1.2
4.67 + 0.33	9.25 + 5.12	4.33 + 1.76	3.25 + 1.6	1.25 + 0.63	2 + 0.58	2.25 + 0.48	0.75 + 0.48
4.67 + 4.18	11.75 + 6.02	6.67 + 3.18	1.75 + 0.85	2.5 + 1.32	1.5 + 1.19	1.25 + 0.95	1 + 0
3.67 + 1.67	4.5 + 2.5	4 + 1.87	3.67 + 0.67	3.75 + 0.85	0.75 + 0.48	0.25 + 0.25	0.5 + 0.29
0.75 + 0.75	3.5 + 1.19	11.33 + 5.36	8.25 + 3.22	3 + 1	2.5 + 1.19	0.5 + 0.29	0.5 + 0.29
5 + 1.87	7.5 + 3.3	10 + 5.9	3.75 + 1.25	2.75 + 1.25	4.5 + 1.66	3.25 + 2.29	0.5 + 0.29
6 + 2.42	13.25 + 6.76	15 + 7.52	6.25 + 2.14	5.75 + 0.75	4 + 1.08	1.25 + 0.63	0.75 + 0.48
0.5 + 0.29	3.5 + 1.71	3 + 2	1 + 0.41	0.5 + 0.29	1 + 0.58	0	0
12.75 + 5.57	27.5 + 17.56	7.75 + 2.66	19.33 + 10.53	8.25 + 2.66	11.5 + 3.66	2.25 + 0.75	2.5 + 1.04
7.25 + 3.01	13.5 + 4.79	16.75 + 6.1	11 + 3.39	6.75 + 2.95	12.5 + 7.01	3.5 + 3.18	2 + 1.08
13.75 + 4.87	17.75 + 4.23	33.5 + 6.01	8.5 + 4.5	22.75 + 4.61	11 + 3.08	17.25 + 2.39	4.5 + 2.84
6 + 1.35	6.33 + 2.19	12.33 + 1.67	4 + 1	8.5 + 3.38	3.33 + 0.88	7 + 1.22	3.67 + 2.19
5.75 + 1.75	5.5 + 0.96	3.5 + 1.44	3 + 0.41	3 + 1.08	1.75 + 0.75	3.75 + 2.25	0.25 + 0.25
1.67 + 0.67	1.75 + 0.48	4.5 + 1.19	3 + 3	4 + 1.47	2.25 + 0.48	2 + 1.35	0
3 + 1	5 + 0.58	3.75 + 1.49	5 + 2.65	4 + 1.22	3.25 + 0.95	1 + 0.58	0
5 + 1.35	6.25 + 1.75	5.75 + 0.85	5.33 + 0.67	2.25 + 0.63	3.25 + 1.31	1.25 + 0.63	0
3.67 + 0.88	4.75 + 0.48	6.25 + 0.85	8 + 1.78	4.75 + 0.25	4.33 + 0.33	1.25 + 0.48	0

percentage of areas covered by “vegetation” vs “artificial surfaces”) at a 300 m and 3 km radius areas. NA=not available.
