# Supplementary material for: Spatial and Temporal Hot Spots of Aedes albopictus Abundance inside and outside a South European Metropolitan Area
Source: PLoS Negl Trop Dis. 2016 Jun 22;10(6):e0004758. doi: 10.1371/journal.pntd.0004758 (PMC4917172; doi:10.1371/journal.pntd.0004758)
Supplement: S2 Table — Rainfall variables were modelled in turn either non-linearly or linearly with the inclusion of a Day of Year (DoY) smoother in each model. Temperature (T) variables were modelled in turn non-linearly (GDD = Growing Degree Days; LST = Land Surface Temperature). For T variables, the DoY smoother was not included due to high collinearity (i.e. concurvity) between T and DoY. s() denotes the smoother term. (DOCX) [file pntd.0004758.s002.docx]

**Table S2: Result of Generalized Additive Mixed Models (GAMMs) of time-dependent climatic predictors during the whole sampling season.** Rainfall variables were modelled in turn either non-linearly or linearly with the inclusion of a Day of Year (DoY) smoother in each model. Temperature (T) variables were modelled in turn non-linearly (GDD=Growing Degree Days; LST=Land Surface Temperature). For T variables, the DoY smoother was not included due to high collinearity (i.e. concurvity) between T and DoY. s() denotes the smoother term.

| **Model** | **Rainfall Variables** | **AIC** | **Delta AIC** | **Intercept** | **Coeff** | **Statistic coeff** | **Statistic smoother** |
| --- | --- | --- | --- | --- | --- | --- | --- |
| GAMM-5 | s(Rainfall Lag 4) | 6717.86 | 0.00 | 1.70 | - | - | <0.0001 |
| GAMM-3 | s(Rainfall Lag 2) | 6733.64 | 15.78 | 1.71 | - | - | <0.0001 |
| GAMM-1 | s(Rainfall Lag 0) | 6738.16 | 20.29 | 1.72 | - | - | <0.0001 |
| GAMM-2 | s(Rainfall Lag 1) | 6746.40 | 28.54 | 1.72 | - | - | <0.0001 |
| GAMM-4 | s(Rainfall Lag 3) | 6755.81 | 37.95 | 1.71 | - | - | <0.0001 |
| GAMM-6 | Rainfall Lag 0 + s(DoY) | 6501.57 | 0.00 | 1.62 | -0.06 | 0.06 | <0.0001 |
| GAMM-10 | Rainfall Lag 4 + s(DoY) | 6502.98 | 1.42 | 1.62 | 0.08 | 0.13 | <0.0001 |
| GAMM-8 | Rainfall Lag 2 + s(DoY) | 6504.80 | 3.23 | 1.62 | -0.03 | 0.50 | <0.0001 |
| GAMM-7 | Rainfall Lag 1 + s(DoY) | 6505.21 | 3.64 | 1.62 | 0.00 | 0.92 | <0.0001 |
| GAMM-9 | Rainfall Lag 3 + s(DoY) | 6505.21 | 3.64 | 1.62 | 0.00 | 0.94 | <0.0001 |
|  | **Temperature Variables** |  |  |  |  |  |  |
| GAMM-15 | s(LST Lag 4) | 6543.76 | 0.00 | 1.63 | - | - | <0.0001 |
| GAMM-14 | s(LST Lag 3) | 6543.79 | 0.03 | 1.63 | - | - | <0.0001 |
| GAMM-11 | s(LST Lag 0) | 6546.24 | 2.48 | 1.63 | - | - | <0.0001 |
| GAMM-13 | s(LST Lag 2) | 6553.69 | 9.93 | 1.64 | - | - | <0.0001 |
| GAMM-19 | s(GDD) | 6556.81 | 13.05 | 1.64 | - | - | <0.0001 |
| GAMM-16 | s(LST Min) | 6557.84 | 14.08 | 1.64 | - | - | <0.0001 |
| GAMM-12 | s(LST Lag 1) | 6563.77 | 20.01 | 1.64 | - | - | <0.0001 |
| GAMM-17 | s(LST Max) | 6564.95 | 21.19 | 1.64 | - | - | <0.0001 |
| GAMM-20 | s(Accumulated GDD) | 6689.23 | 145.46 | 1.69 | - | - | <0.0001 |
| GAMM-21 | s(Bounded GDD) | 6690.06 | 146.30 | 1.69 | - | - | <0.0001 |
| GAMM-18 | s(Temperature Range) | 6755.43 | 211.66 | 1.72 | - | - | <0.0001 |
|  | **Day of Year Variable** |  |  |  |  |  |  |
| GAMM-22 | s(DoY) | 6510.73 | 0 | 1.62 | - | - | <0.0001 |
