# Supplementary material for: Spatial and Temporal Hot Spots of Aedes albopictus Abundance inside and outside a South European Metropolitan Area
Source: PLoS Negl Trop Dis. 2016 Jun 22;10(6):e0004758. doi: 10.1371/journal.pntd.0004758 (PMC4917172; doi:10.1371/journal.pntd.0004758)
Supplement: S3 Table — (GDD = Growing Degree Days; LST = Land Surface Temperature). (DOCX) [file pntd.0004758.s003.docx]

**Table S3:** **Result of Generalized Linear Mixed Models (GLMMs) of time-dependent climatic predictors during the two high *Aedes albopictus* abundance phases.** (GDD=Growing Degree Days; LST=Land Surface Temperature).

| **Phase-1** | | | | | **Phase-2** | | | | |
| --- | --- | --- | --- | --- | --- | --- | --- | --- | --- |
| **Model** | **Rainfall Variable** | **AIC** | **Delta AIC** | **Statistic coeff** | **Model** | **Rainfall Variable** | **AIC** | **Delta AIC** | **Statistic coeff** |
| GLMM-2 | Rainfall Lag 1 | 3277.56 | 0.00 | 0.0103 | GLMM-5 | Rainfall Lag 4 | 2295.44 | 0 | <0.0001 |
| GLMM-3 | Rainfall Lag 2 | 3280.32 | 2.76 | 0.0573 | GLMM-1 | Rainfall Lag 0 | 2309.06 | 13.62 | 0.0011 |
| GLMM-4 | Rainfall Lag 3 | 3281.64 | 4.08 | 0.1347 | GLMM-4 | Rainfall Lag 3 | 2316.4 | 20.96 | 0.0797 |
| GLMM-1 | Rainfall Lag 0 | 3282.80 | 5.24 | 0.3117 | GLMM-3 | Rainfall Lag 2 | 2317.12 | 21.68 | 0.1209 |
| GLMM-5 | Rainfall Lag 4 | 3283.14 | 5.58 | 0.4058 | GLMM-2 | Rainfall Lag 1 | 2317.74 | 22.3 | 0.1869 |
|  | **Temperature Variable** |  |  |  |  | **Temperature Variable** |  |  |  |
| GLMM-7 | LST Lag 1 | 3264.76 | 0.00 | <0.0001 | GLMM-6 | LST Lag 0 | 2310.74 | 0 | 0.0027 |
| GLMM-15 | Accumulated GDD | 3267.50 | 2.74 | <0.0001 | GLMM-12 | LST Max | 2313.54 | 2.8 | 0.0140 |
| GLMM-11 | LST Min | 3267.52 | 2.76 | <0.0001 | GLMM-7 | LST Lag 1 | 2314.66 | 3.92 | 0.0263 |
| GLMM-16 | Bounded GDD | 3267.98 | 3.22 | 0.0001 | GLMM-14 | GDD | 2314.72 | 3.98 | 0.0277 |
| GLMM-8 | LST Lag 2 | 3269.76 | 5.00 | 0.0002 | GLMM-8 | LST Lag 2 | 2317.2 | 6.46 | 0.1288 |
| GLMM-9 | LST Lag 3 | 3271.74 | 6.98 | 0.0007 | GLMM-13 | Temperature Range | 2317.22 | 6.48 | 0.1333 |
| GLMM-10 | LST Lag 4 | 3273.94 | 9.18 | 0.0023 | GLMM-11 | LST Min | 2318.24 | 7.5 | 0.2654 |
| GLMM-14 | GDD | 3275.82 | 11.06 | 0.0042 | GLMM-9 | LST Lag 3 | 2319.08 | 8.34 | 0.5260 |
| GLMM-6 | LST Lag 0 | 3277.76 | 13.00 | 0.0128 | GLMM-10 | LST Lag 4 | 2319.4 | 8.66 | 0.7785 |
| GLMM-12 | LST Max | 3280.32 | 15.56 | 0.0591 | GLMM-16 | Bounded GDD | 2319.46 | 8.72 | 0.8876 |
| GLMM-13 | Temperature Range | 3282.88 | 18.12 | 0.3390 | GLMM-15 | Accumulated GDD | 2319.46 | 8.72 | 0.8638 |
