# Supplementary figures and images for: Spatial and Temporal Hot Spots of Aedes albopictus Abundance inside and outside a South European Metropolitan Area
Source: PLoS Negl Trop Dis. 2016 Jun 22;10(6):e0004758. doi: 10.1371/journal.pntd.0004758 (PMC4917172; doi:10.1371/journal.pntd.0004758)

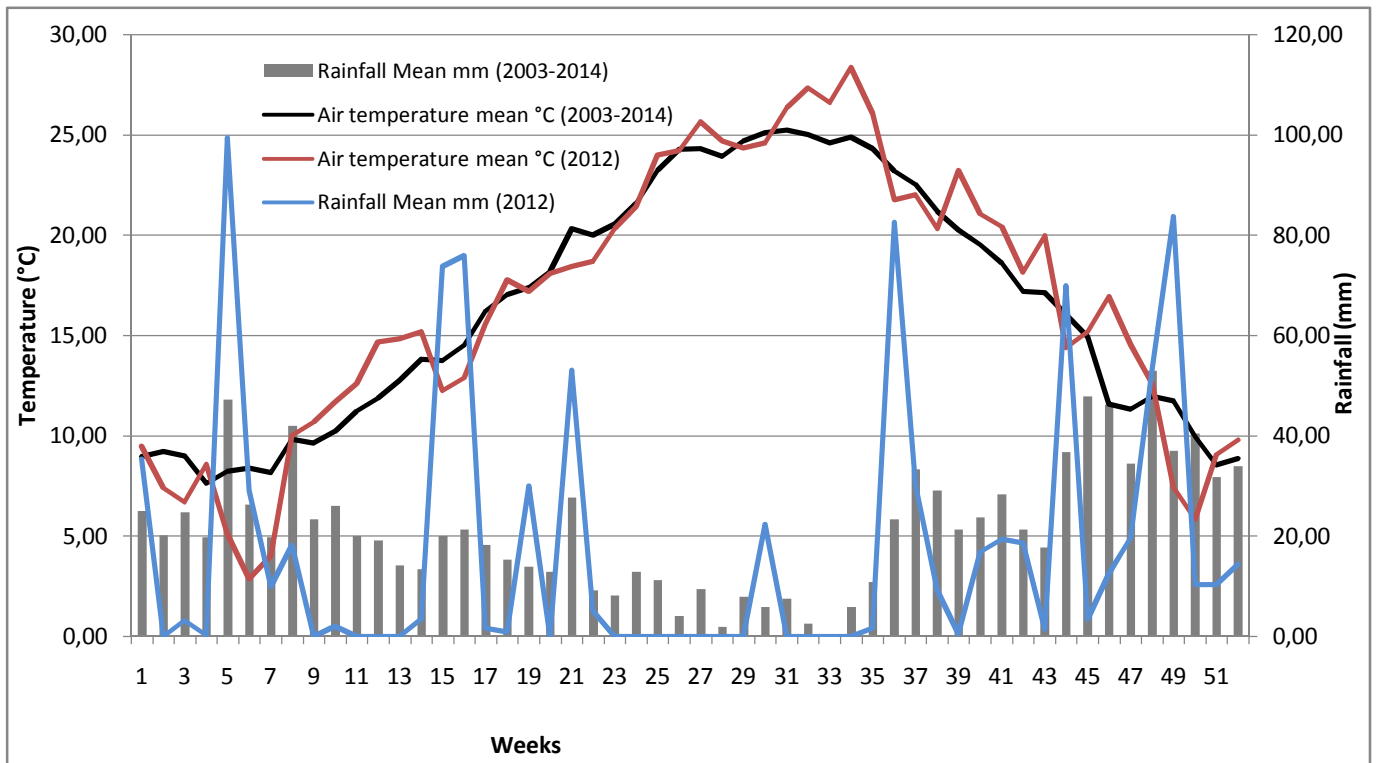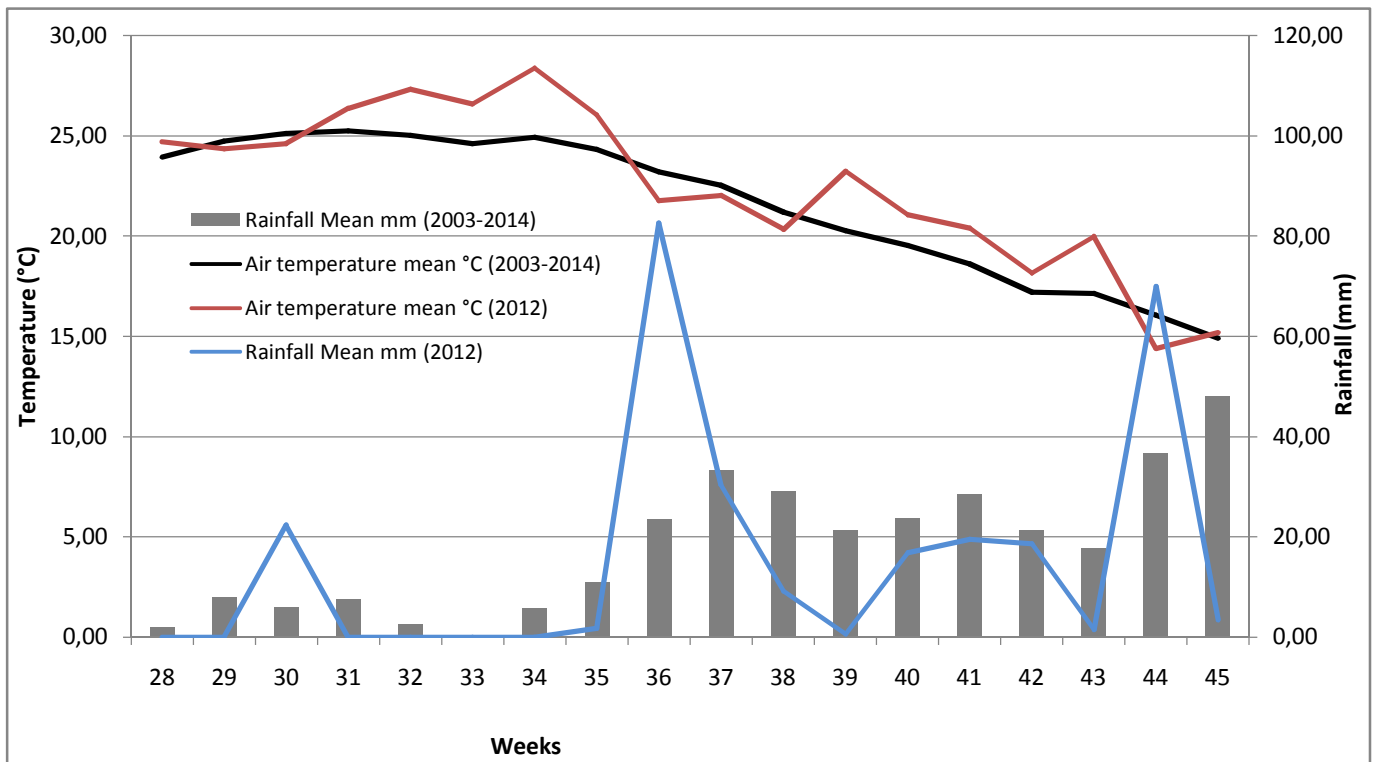

Supplement: S1 Fig — Data collected by the Hydrographic Service of Regione Lazio and disseminated through the hydrographic annals (http://www.idrografico.roma.it/annali). Meteorological sampling stations of Roma Sud. Upper panel: whole year data, Lower panel: highlight week 28–45 from whole year data. (PDF) [file pntd.0004758.s004.pdf]
